# Nanoscale distribution of Bi atoms in InP_1−x_Bi_x_

**DOI:** 10.1038/s41598-017-12075-2

**Published:** 2017-09-25

**Authors:** Liyao Zhang, Mingjian Wu, Xiren Chen, Xiaoyan Wu, Erdmann Spiecker, Yuxin Song, Wenwu Pan, Yaoyao Li, Li Yue, Jun Shao, Shumin Wang

**Affiliations:** 1 State Key Laboratory of Functional Materials for Informatics, Shanghai Institute of Microsystem and Information Technology, CAS, 865 Changning Road, Shanghai, 200050 China; 2 Institute of Micro- and Nanostructure Research & Center for Nanoanalysis and Electron Microscopy (CENEM), Department of Materials Science, Universität Erlangen-Nürnberg, Cauerstraße 6, D-91058 Erlangen, Germany; 3 Paul-Drude-Institut für Festkörperelektronik, Hausvogteiplatz 5-7, D-10117 Berlin, Germany; 4 National Laboratory for Infrared Physics, Shanghai Institute of Technical Physics, CAS, 500 Yutian Road, Shanghai, 200083 China; 5 Department of Microtechnology and Nanoscience, Chalmers University of Technology, 41296 Gothenburg, Sweden

## Abstract

The nanoscale distribution of Bi in InPBi is determined by atom probe tomography and transmission electron microscopy. The distribution of Bi atoms is not uniform both along the growth direction and within the film plane. A statistically high Bi-content region is observed at the bottom of the InPBi layer close to the InPBi/InP interface. Bi-rich V-shaped walls on the (−111) and (1–11) planes close to the InPBi/InP interface and quasi-periodic Bi-rich nanowalls in the (1–10) plane with a periodicity of about 100 nm are observed. A growth model is proposed to explain the formation of these unique Bi-related nanoscale features. These features can significantly affect the deep levels of the InPBi epilayer. The regions in the InPBi layer with or without these Bi-related nanostructures exhibit different optical properties.

## Introduction

Dilute bismide is a new member of the III-V compound semiconductor family. Since GaAsBi was first successfully grown by metal-organic vapor phase epitaxy (MOVPE) in 1998^1^ and molecular beam epitaxy (MBE) in 2003^2^, a lot of benefits have been obtained from the dilute bismides. Incorporating every 1% Bi atoms can produce bandgap reduction for GaAsBi, GaSbBi, InAsBi, InSbBi and InPBi of 88meV^3^, 35meV^4^, 55meV^5^, 36meV^6^ and 106meV^7^, respectively, which effectively extends light emitting wavelength of traditional III-V compounds. The large atomic size of Bi also increases the spin-orbit splitting energy of the matrix material which could inhibit Auger recombination involving holes in the valence band and the spin-orbit splitting band, as well as reduce the sensitivity of the bandgap to ambient temperature^8,9^. Meanwhile, Bi can act as a surfactant during material growth^10–12^. Owing to these properties, dilute bismide has drawn interest for its application in near/mid-infrared lasers, solar cells, and spintronic devices^13–16^. InPBi was first successfully realized by MBE in 2013^17^ after the theoretical prediction by Berding *et al*.^18^. Compared with other dilute bismides, InPBi shows the largest bandgap reduction and exhibits a broad and strong photoluminescence (PL) spectrum at 1.4–2.7 μm, which renders InPBi a potential material for the fabrication of super-luminescent diodes applied in optical coherence tomography(OCT).

Phase separation can occur in dilute bismides as an uncontrolled consequence of incorporated Bi atoms because of the generally large miscibility gap between Bi and III-V counterparts. Atomic ordering^19,20^, nanoscale concentration modulation^21–23^ or even cluster formation^24^ have been reported in the prototype materials GaAsBi and InAsBi. Most studies on InPBi have focused on epitaxial growth optimization, optical properties and electronic properties^7,25–29^ without sufficient knowledge of the microstructures. Detailed nanoscale structural analysis and the relation between the nanostructures to the physical properties have yet to be conducted. To obtain more physical insights of the material property and promote device applications, understanding the structure at the nanoscale level and coupling it to physical properties are necessary.

(Scanning) transmission electron microscopy (S/TEM) has been widely used in nanoscale phase separation^19–24^, delivering rich microstructural information from the nanometer scale to the atomic scale by using various types of contrast mechanisms. However, in the normal setup, S/TEM only yields projected information over a thickness of typically a few tens to about a hundred nanometers. Atom probe tomography (APT) can provide three-dimensional (3D) recovery of the atomic distribution of measured material and is highly sensitive to chemical composition up to tens of ppm level in favorable cases^30^. However, owing to the limitation of spatial aberrations for different atoms, the apex radius of the needle-shaped sample measured by APT can be the maximum at 200 nm^31^. APT and S/TEM can thus complement each other and provide quantitative and statistically relevant information in 3D.

In the present study, S/TEM and APT are applied to investigate the distribution of Bi atoms both out-of-plane and in-plane in InPBi. Anomalous nanoscale distributions of Bi atoms, such as V-shaped features at the epitaxial interface and Bi-rich nanowalls quasi-periodically modulated along the [1–10] direction are identified through a combination of TEM and APT. The origins of these nanostructures are discussed. Furthermore, the optical property correlated to the nanoscale distribution of Bi atoms is also discussed in detail.

## Results

**Figure 1:**
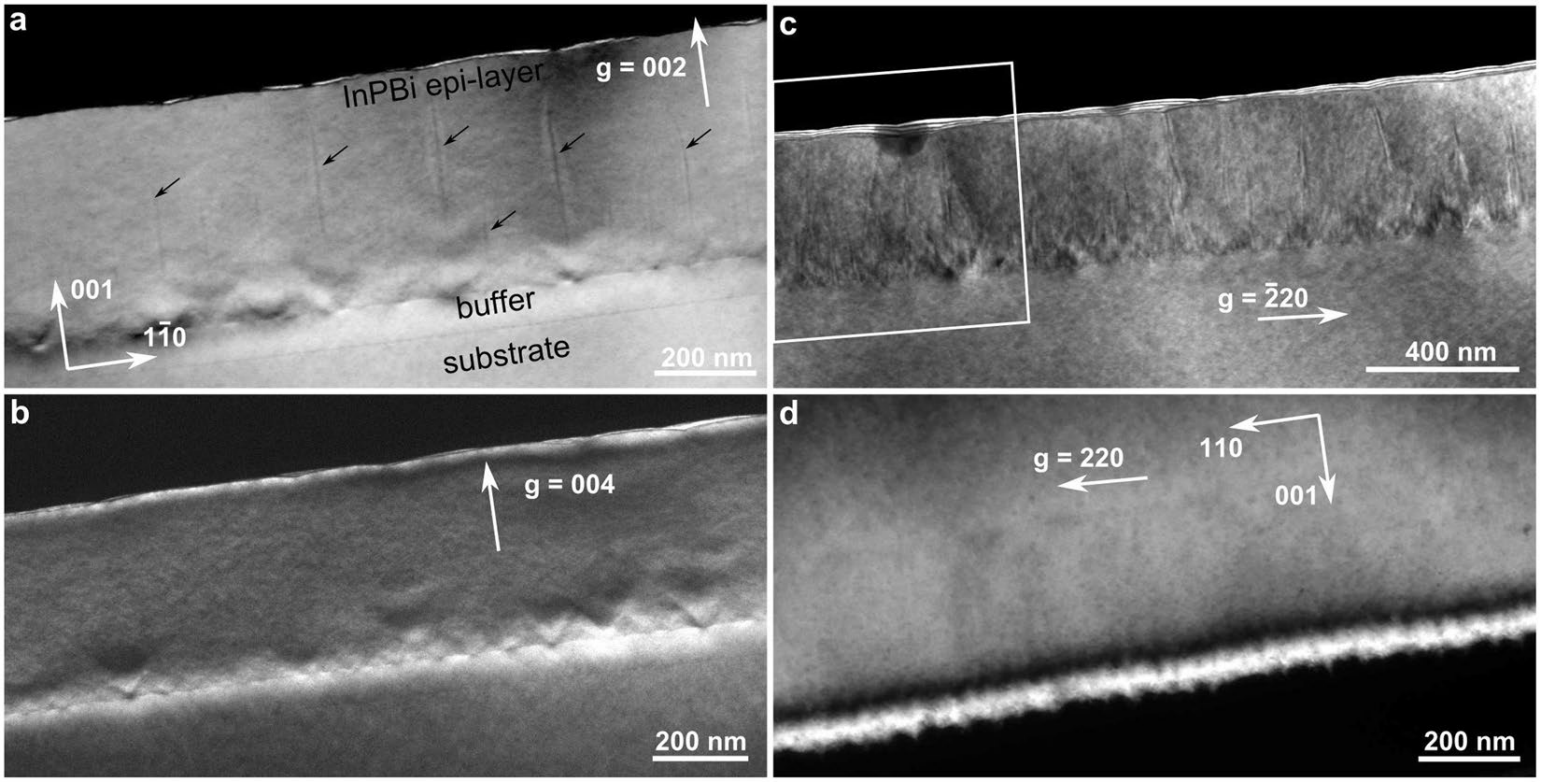
Cross-section of the dark-field TEM images of Specimen A under the two-beam condition with *g* = 002 (**a**), 004 (**b**) and $\bar{2}20$ (**c)** close to the [110] zone axis and with *g* = 220 close to the [$\bar{1}10$] zone axis. The V-shaped feature at the bottom of the InPBi layer close to the InP/InPBi interface and the stripes in the InPBi layer are clearly observed in (**a**), which corresponds to the Bi-rich regions. The V-shaped features are inclined to the interface at about 55°, indicating that they are in the {111} plane, whereas the Bi-rich columns stretching along the growth direction from the center of the V-shaped feature are quasi-periodic with a periodicity of about 100 nm. The box mark in (**c**) indicates the position where the STEM-EDX spectrum image maps of Fig. 2 were acquired. In the perpendicular [1–10] zone axis, no V-shaped features and vertical stripes are observed.

Four specimens (Specimens A-D) were cut from the same InP_1−x_Bi_x_ sample with Bi content of 2.3%. Cross-section TEM and STEM-EDX were performed to characterize Bi-related nanostructures. Figure 1 shows the dark-field TEM micrographs of Specimen A under three two-beam conditions. The image contrast provides local chemical and strain information depending on the applied conditions. These imaging techniques have been successfully applied to study the lateral composition modulation (LCM) in a short-period strained-layer superlattice^32^. For zincblende AB_1−x_C_x_ alloys, with *g* = 002 condition, the contrast is mainly due to the difference in atomic scattering factor. Thus, the image contrast is chemically sensitive to the alloying content *x*, at least to the qualitative level^33^. In the current study, the dark contrast is attributed to Bi-rich regions. V-shaped features appear at the interface of the InPBi and InP buffer layer and dark stripes stretch toward the surface from the center of the V-shaped feature, as shown in Fig. 1(a). Shallow pits on the surface exist and are correlated to the dark stripes. The stripes are arranged in a quasi-periodic manner with a periodicity of about 100 nm. The two slanted sides of the Bi-rich V-shaped feature are inclined to the interface at about 55°, which matches the {111} planes. The image shown in Fig. 1(b) with *g* = 004 is sensitive to epitaxial strain in the out-of-plane, i.e., growth direction. A high contrast at the InPBi/InP interface is clearly observed, indicating strong epitaxial strain. In addition, V-shaped contrast features are visible close to the interface region. Despite the V-shaped features, the InPBi epilayer is completely coherent with respect to the InP buffer layer (i.e., no interfacial misfit dislocation is observed) over the whole observable area of about a few tens of micrometers (shown in the Supporting Information). Figure 1(c) presents a dark-field image under a two-beam condition with *g* = $\bar{2}20$. The image contrast under this condition provides information about the in-plane strain. In addition to the V-shaped features at the interface, the vertical stripes with a quasi-periodic arrangement are prominent and clearly visible. Figure 1(a–c) present images obtained close to the [110] zone axis and Fig. 1(d) shows the image acquired in the perpendicular direction close to the [1–10] zone axis, with *g* = 220 under the two-beam condition. Contrary to Fig. 1(c), no V-shaped feature and periodic strip contrast is observed. The vertical stripes are assigned to Bi-rich nanowalls, as subsequently confirmed and quantitatively evaluated by APT.

**Figure 2:**
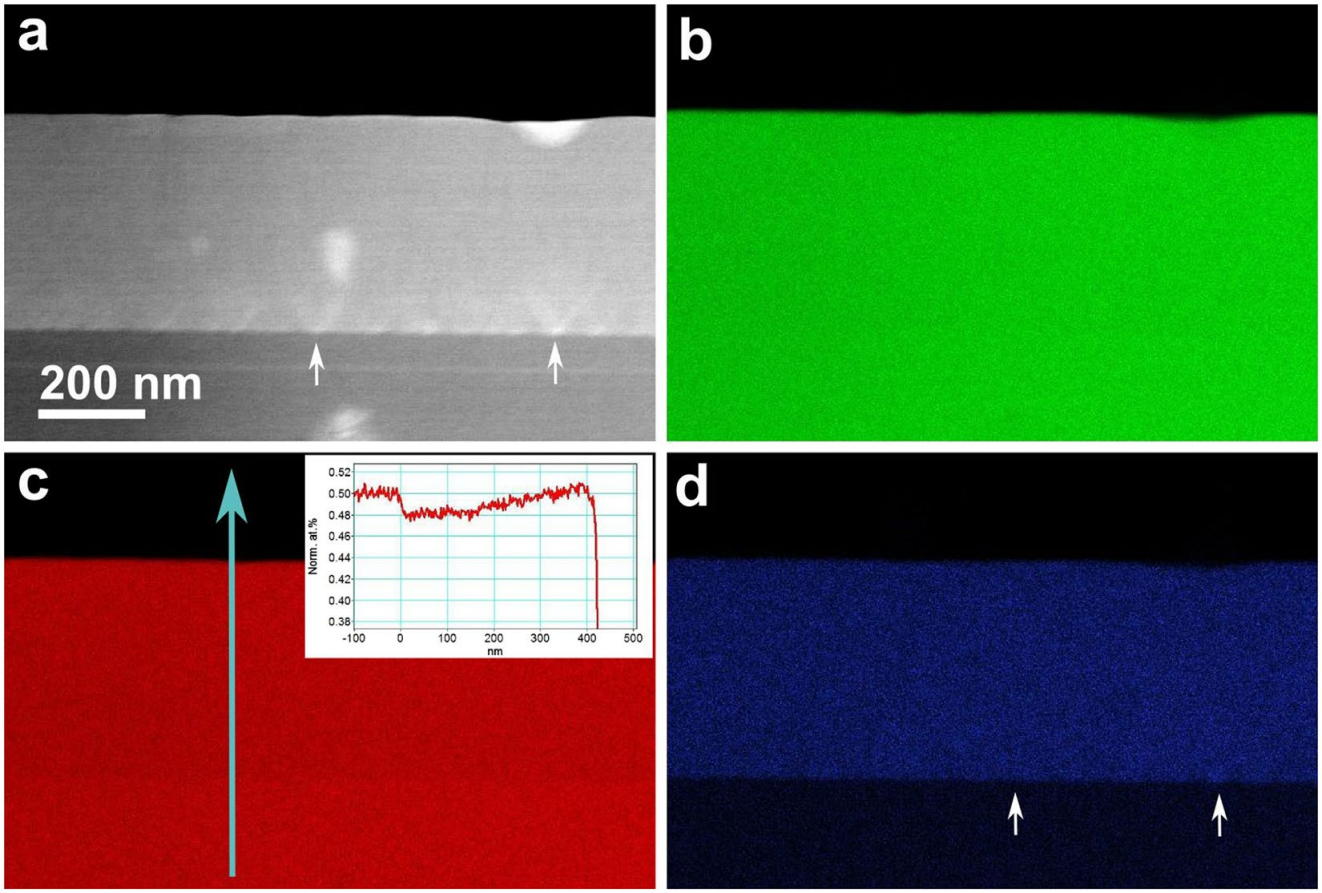
STEM-EDX spectrum image map. (**a**) The medium angle annular dark-field (MAADF)-STEM image, which provides a mixed Z- and strain contrast. Bright spots appear in the middle and at the bottom, which are due to unintentional carbon contamination during TEM sample preparation. (**b**–**d**) Background subtracted EDX net intensity maps of In, P and Bi. A reduced P atomic ratio of about 2% at the onset of the InPBi interface is clearly seen in the inset of (**c**). Slight Bi-rich areas denoted by a white arrow in (**d**) correlates to the bottom of the V-shaped features in (**a**). Bi-rich stripes are not observed at a reasonable acquisition time (cf. Methods section).

STEM-EDX spectrum imaging maps were acquired at the location, as marked in Fig. 1(c) and the results are presented in Fig. 2. In Fig. 2(a), the epilayer interface and the V-shaped features are revealed by medium angle annular dark-field (MAADF)-STEM imaging, which provides a mixed Z- and strain contrast. Under this condition, the Bi-rich stripes are not visible, as clearly evidenced in Fig. 1(c). In Fig. 2(b), the In map appears considerably homogeneous from the substrate to the epilayer. In Fig. 2(c), a sharp interface at the epilayer appears in the P map. A line profile (with an integration width of 300 pixels) extracted from the P map along the growth direction is shown as the inset in Fig. 2(c). The background subtracted EDX net intensity of P K-lines is normalized to the intensity at the substrate to yield the expected ratio of 50 at.% of InP. In this manner, a reduction of about 2 at.% of P occurs, as shown in the profile, at the onset of the epilayer interface. Along the growth direction, the P atomic ratio gradually recovers to about 50%. In the Bi map, seemingly high-Bi concentration spots are roughly visible. These spots are correlated to the location of the bottom of the V-shaped features. The Bi content in the InPBi epilayer is evaluated at 2–4%, which agrees with the results of secondary ion mass spectroscopy (SIMS).

**Figure 3:**
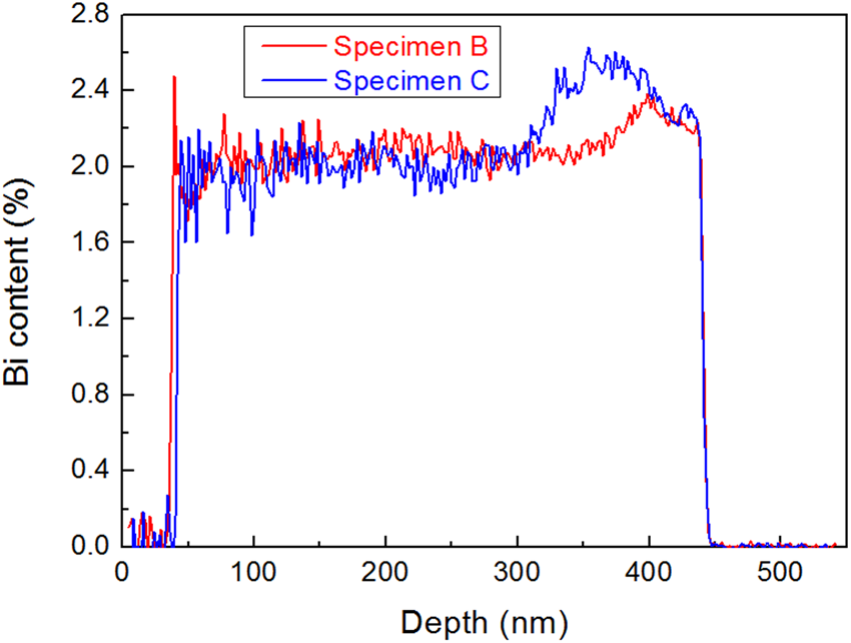
Depth profile of Bi atoms measured by APT. For both specimens, a Bi concentration peak is observed at the bottom of the InPBi layer close to the InPBi/InP interface, with Specimens B and C reaching peak concentrations of 2.32% and 2.56%, respectively.

Bi-related structures, including the Bi-rich V-shaped features at the InPBi/InP interface and Bi-rich nanowalls are deduced based on the observation from two perpendicular cross-sections observed by TEM. The exact 3D shape of the nanowall, particularly the chemical content, are quite challenging to obtain from the aforementioned results. APT was conducted to obtain a 3D distribution of Bi atoms. Figure 3 presents a plot of the average depth profile of Bi atom counts measured by APT, which, for STEM-EDX, seems incapable (cf. Fig. 2) at such a low Bi concentration. In the APT plot, the first 40 nm is a GaAs protective cap sputtered in the sample preparation; no significant Bi signal is detected. The overall Bi count exhibits violent fluctuations, which decrease with depth, near the surface. This fluctuation is attributed to the increase in sampling volume when sweeping from the pinnacle to the bottom of the sample tip. The average count of Bi atoms remains almost unchanged and is centered at around 2.08% in the 150–370 nm region for Specimen B and 120–300 nm region for Specimen C. For Specimen B, an increase in Bi concentration occurs at the bottom of the InPBi layer, with a thickness of 40 nm, above the interface. The Bi content reaches a peak of about 2.32% and decreases to about 2.2% at the InPBi/InP interface. Similar results are also found in Specimen C with a wider rising region and a higher Bi peak content of about 2.56%.

**Figure 4:**
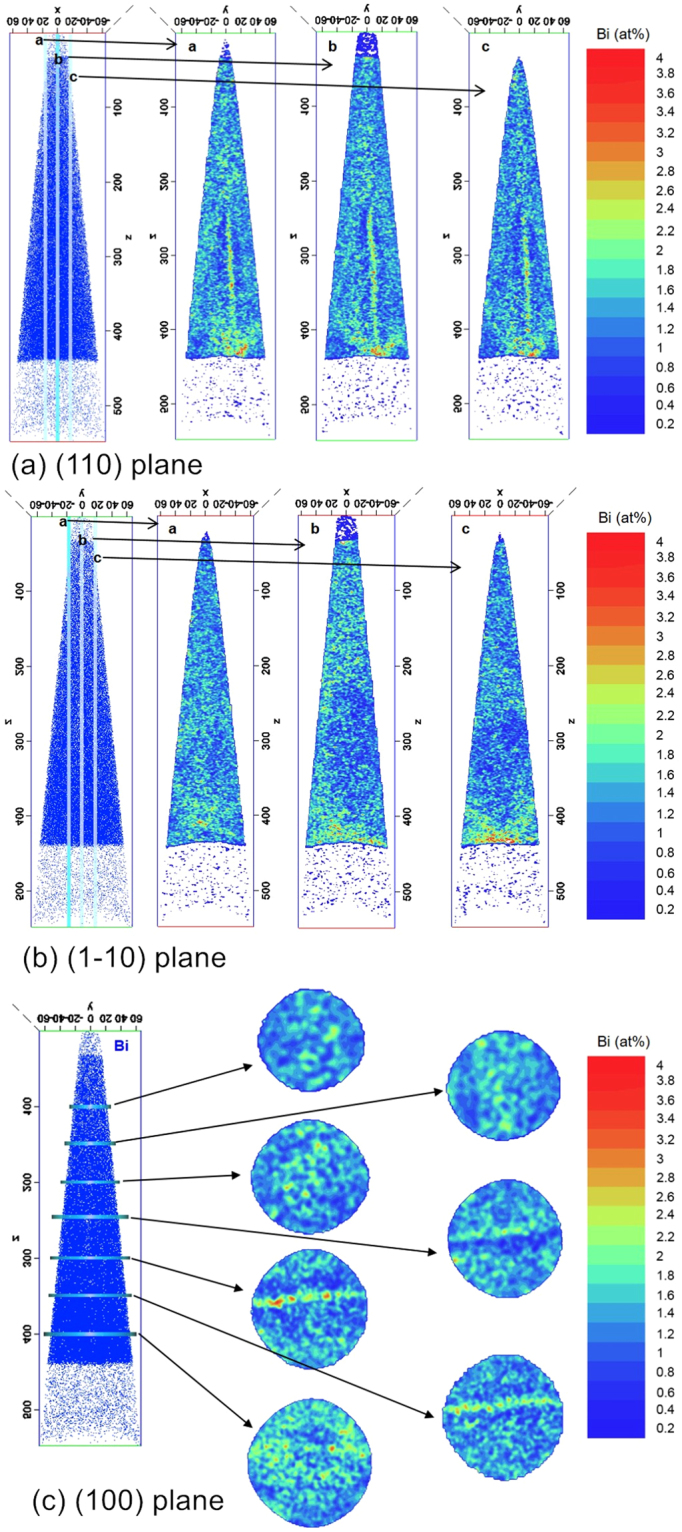
Cross-section representation of the 3D-reconstructed APT dataset of the Bi atom distribution of Specimen B: projected onto the (**a**) (110) plane, (**b**) (1–10) plane and (**c**) (100) plane with the *x*, *y*, and *z* axes representing the [110], [1–10] and [001] direction, respectively. A Bi-rich V-shaped feature clearly appears in (**a**) but not in (**b**) because of the asymmetry of the InP lattice. A Bi-rich column stretches from the center of the V-shaped feature toward the surface and ending in the InPBi layer. Combining (**a**) and (**c**), we conclude that this Bi-rich V-shaped feature is a Bi-rich V-wall, and the Bi-rich column is a Bi-rich nanowall in the (1–10) plane.

Figure 4 shows the (110), (1–10) and (100) planes of Specimen B at different intersections, with the *x, y* and *z* directions defined as the [110], [1–10] and [001] direction, respectively. The [001] direction represents the growth direction. Each plane consists of InPBi with a thickness of 5 nm. The Bi distribution is non-uniform and reveals interesting patterns. A high-Bi content region in the V-shaped feature is also observed by TEM at the bottom of the InP_1−x_Bi_x_ layer in the (110) plane and in some of the (1–10) planes (not as clear as in the (110) plane). The highest Bi atomic count is 4%, denoted by red spots corresponding to a local Bi content of 8% in the InP_1−x_Bi_x_ thin film. The V-shaped feature spans, on the average, 73 nm and 33 nm along the [1–10] and [001] directions, respectively. The Bi concentration decreases along the two axes.

**Figure 5:**
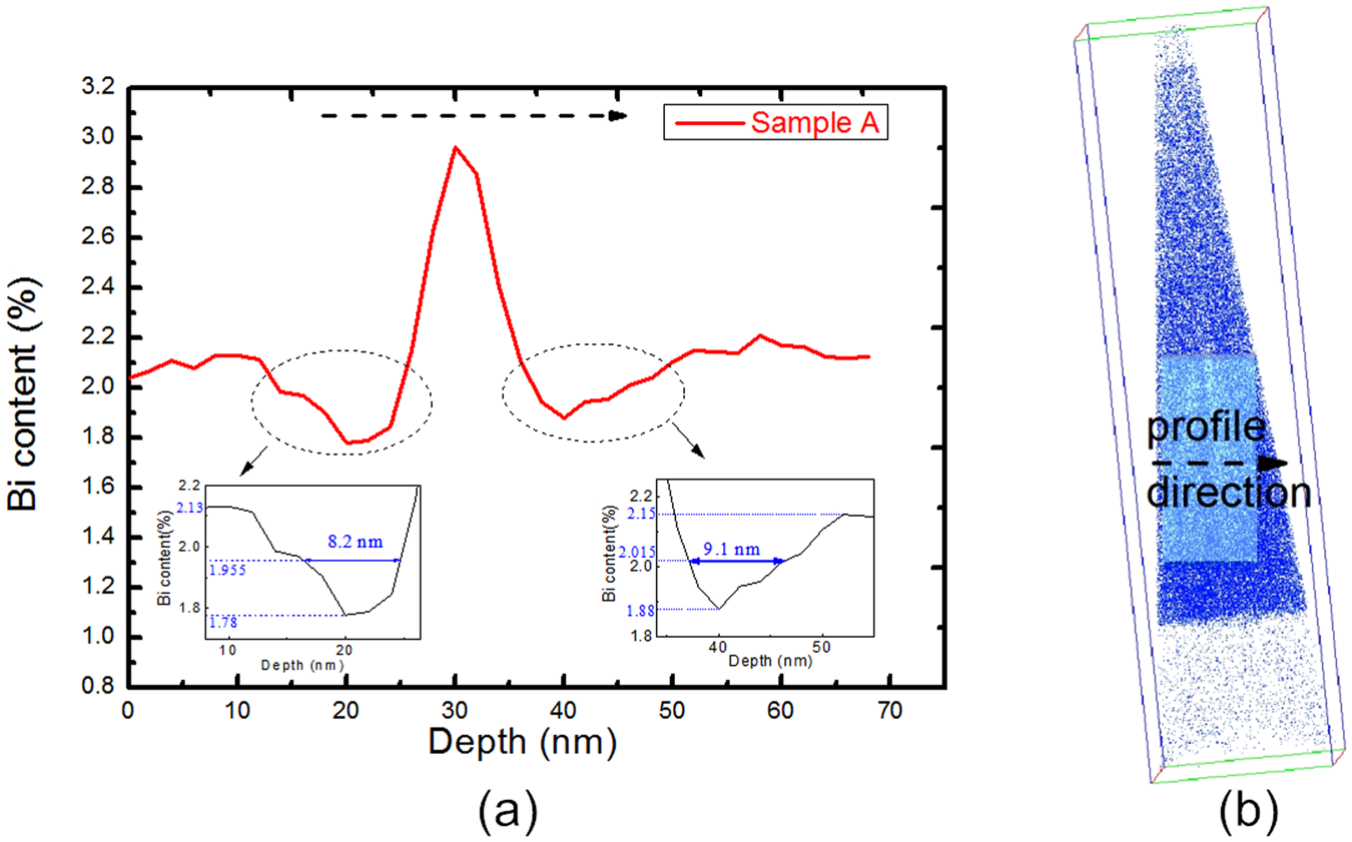
Bi depth profile (**a**) in the arrow direction in (**b**). A Bi concentration peak exists in the profile direction with two depletion regions of Bi on both sides of the Bi concentration peak, with the details partially enlarged in the insets in (**a**).

From the center of the V-shaped feature toward the surface, a Bi-rich column consistent with the cross-section TEM image exists only on the (110) plane. On the basis of the different projections of the 3D volume, we deduce that the Bi-rich V-shaped feature is a Bi-rich V-shaped plane (V-wall) on the {111} plane and that the Bi-rich nano-column on the (110) plane is a Bi-rich plane (nanowall) of in average 206 nm along the [001] direction and 3~5 nm along the [1–10] direction. Within the nanowall, the Bi concentration is also non-uniform, as evidenced by red spots observed in the (100) plane images. A region with a deficiency in Bi content exists on both sides of the Bi-rich plane. An example of a Bi concentration profile is illustrated in Fig. 5. The Bi-rich nanowall ends approximately at half of the InPBi film thickness. The upper half reveals a relatively uniform Bi distribution. Consistent with the TEM images, the APT results unambiguously identify the Bi-rich V-shaped feature and nano-columns observed in TEM as V-shaped nanowalls in the (−111) and (1–11) planes and nanowalls in the (1–10) plane.

Figure 5(a) shows a Bi depth profile in InPBi along the arrow direction pointed in Fig. 5(b). A Bi content peak is observed along the arrow direction, with a peak value of 2.96% and a full width at half maximum (FWHM) of 5.5 nm fitted by a Gaussian function. Adjacent to the peak are two Bi content valleys with a valley value of 1.78%, an FWHM of 8.2 nm on the left side and a valley value of 1.88% and an FWHM of 9.1 nm on the right side with partially enlarged details in the insets. The Bi content rises from 2.04% to 2.10% at the first 3.9 nm owing to an increase in the sample volume. The Bi content then fluctuates at about 2.10 ± 0.02% for the next 8.2 nm. Subsequently, the Bi content goes through a valley, a peak and a valley, and ultimately fluctuates at 2.16 ± 0.04%. The Bi enrichment peak draws Bi atoms from nearby, causing a lack of Bi next to the Bi peak.

## Discussion

### Growth model

The unique features of Bi distribution in a nano-column stretching from the center of the V-shaped feature at the InPBi/InP interface toward the surface are observed in TEM. These features are further identified as nanowalls in the (1–10) plane and V-shaped nanowalls in the (−111) and (1–11) planes by APT. However, they have not been observed previously in other dilute bismides. Such appearance may be related to the larger miscibility gap between InBi and InP compared with that of InSb and InAs^18^. In addition, In-Bi is miscible, whereas Ga-Bi exhibits a segregating nature^34,35^.

**Figure 6:**
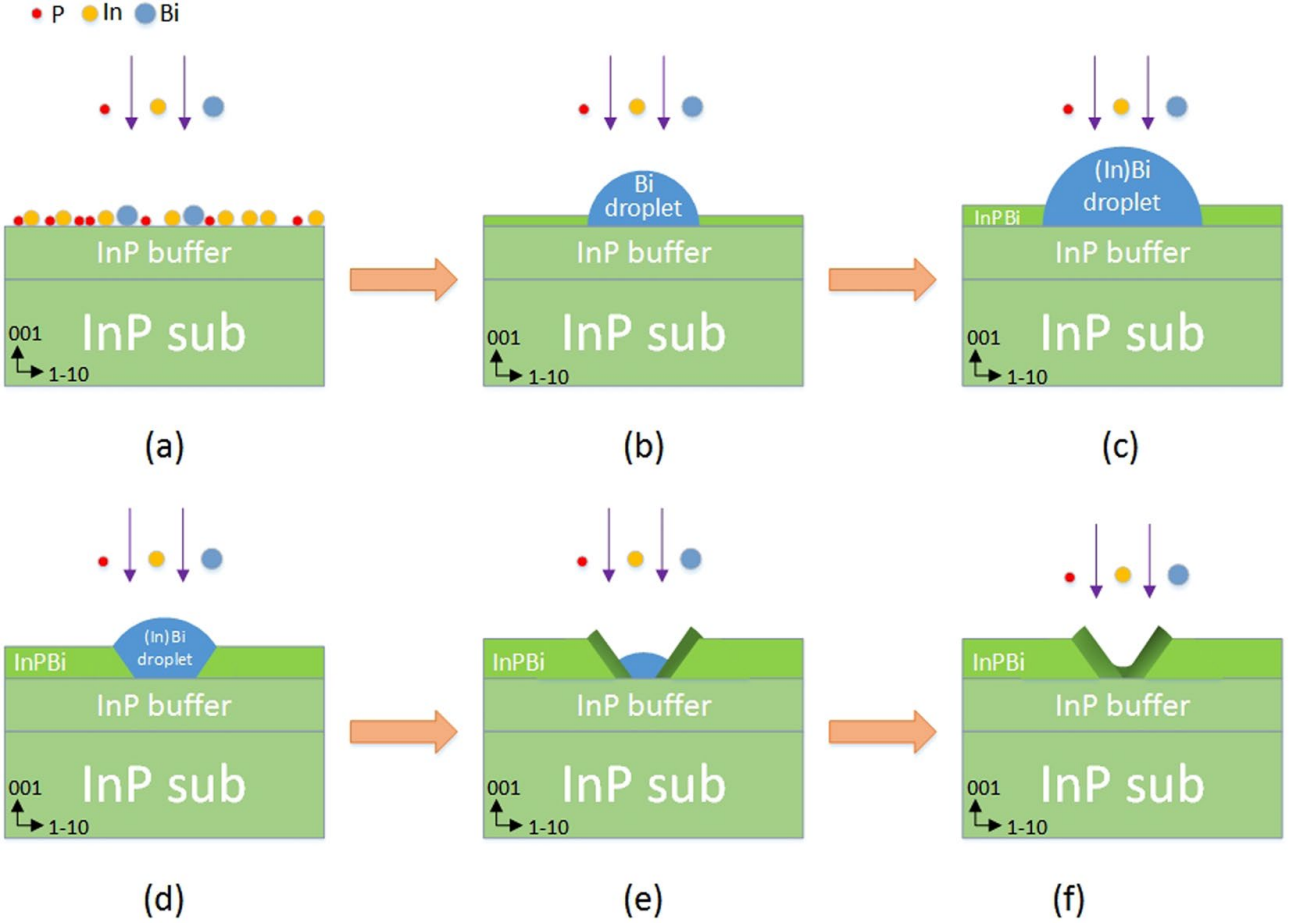
Model of InPBi growth process. Growth proceeds from (**a**) to (**f**). With the Bi droplets formed (**b**) and grown into large (In)Bi droplets (**c**), the growth becomes a combination of molecular beam epitaxy on the InPBi surface and droplet epitaxy at the droplet sites. For droplet epitaxy, nucleation starts at the edge of the droplets and the center of the droplets sinks (**d**). Owing to high Bi concentration in the (In)Bi droplets, the InPBi grown by droplet epitaxy has a Bi concentration gradient with the inner part of a higher Bi content (**e**). In the end, V-shaped nanowalls are formed, as observed by both TEM and APT (**f**).

For V-shaped nanowalls with high Bi content, the process can be interpreted schematically, as shown in Fig. 6. Growth proceeds as shown in Fig. 6(a) to (f). The red, yellow and blue balls represent phosphorus, indium and bismuth atoms, respectively. First, an In effusion cell and a P_2_ cracker shutter were opened to deposit an InP buffer layer with a thickness of 69 nm on the InP substrate at a normal growth temperature. The growth was then interrupted and the growth temperature was decreased. During the initial growth of the InPBi layer, all three atom species diffuse and chemically bond. Owing to the large atomic size of Bi and the weak In-Bi bonding energy with respect to that of In-P, Bi atoms tend to segregate, forming Bi droplets on the surface, as shown in Fig. 6(b). Meanwhile, the P/In flux ratio was maintained at a low level, almost group-III rich and close to the stoichiometric condition to facilitate the incorporation of Bi. The deficiency of P atoms resulted in the accumulation of excess In atoms on the surface. These In atoms can easily diffuse on the surface, forming In droplets; alternatively, it can merge with the existing Bi droplets, forming InBi alloy droplets, as illustrated in Fig. 6(c).

As the growth process occurs, the incoming In and Bi atoms have two alternatives: either to be incorporated, forming chemical bonds as in the case of MBE growth when impinging on the solid InP(Bi) surface, or to be merged in (In)Bi droplets when impinging on (In)Bi liquid droplets. The growth process then becomes a combination of MBE on a solid InPBi surface and droplet epitaxy on (In)Bi droplets.

Reyes *et al*. theoretically simulated and experimentally performed droplet epitaxial growth for GaAs quantum dot growth^36^. They first sputtered 4 MLs of Ga atoms onto a GaAs substrate and then annealed the material for 1 min. Subsequently, they deposited As atoms onto the system. Both theoretical and experimental studies indicated that for Ga droplet epitaxial growth, crystallization started at the bottom edge of Ga droplets and then proceeded inward, forming a volcano-like feature. With the growth proceeding on, the Ga droplets in the “volcano” are consumed and the Ga liquid level gradually sinks. On the basis of this growth model, we consider that in the case of InPBi, crystallization starts at the edge of the droplets when the diffusion of adatoms is limited. The In atoms in the droplets are diffused out of the droplets, forming a volcano-like morphology, similar to the case when the Ga droplets are exposed to As flux. Bismuth is a well-known surfactant for III-Vs growth at high temperatures^10,37^, but a small quantity may be incorporated at low temperatures. In this low temperature droplet epitaxial growth, only a fraction of Bi atoms in the droplets tend to diffuse out to cover the InPBi surface and thus lower the surface energy; the residual Bi atoms remain in the droplets. Thus, the surface level of the liquid within the volcanoes sinks as the growth proceeds, as shown in Fig. 6(d and e). The droplets are eventually depleted, forming a V-shaped InPBi morphology with a Bi concentration gradient from the inner part toward the outer part, as shown in Fig. 6(f). The surface diffusion of In is asymmetric along the two orthogonal [±110] directions and the diffusion length is greater along the [1–10] direction than the [110] direction^38–40^. This implies that In atoms are improbable to cease at the edge of the droplets along the [1–10] direction and the formed asymmetric volcano is elongated along the [1–10] direction. Thus, the cross-section with the V-shaped feature is only observed along the [110] direction, whereas the V-shaped nanowalls in the {111} plane meet only along the [110] direction.

Bi can easily mix with In in liquid form so that the Bi droplets formed on the surface can erode the InP underneath, forming InBi droplets. Although Bi droplets might exist during the later growth of InPBi, the presence of Bi or InBi droplets is not necessary to induce droplet epitaxy, as shown in Fig. 6. At the initial growth of InPBi, no Bi atoms exist in the InP underneath. Droplet epitaxy occurs and the irregular volcano-like surface features can well relax the strain induced by Bi incorporation. However, when Bi is incorporated, thus forming InPBi, the suggested scenario shown in Fig. 6 is unlikely because the effect of Bi segregation attributed to strain can expel the incorporated Bi atoms, diffusing toward the surface. This finding indicates that the peculiar Bi-related nanoscale features can only be observed at the initial stage of growth. During later stage of growth, Bi incorporation and segregation are expected to reach a balance. The relative difference between the Bi content on the growth surface and in the InPBi layer decreases. The presence of Bi droplets does not enhance the local growth rate similar to the droplet epitaxy shown in Fig. 6. Thus, this droplet epitaxial growth only occurs at the InPBi/InP interface.

Theoretical calculation has predicted InPBi with a miscibility gap^18^, providing an opportunity for spinodal decomposition, an evolution from unstable states to stable and (or) metastable states^41^. Spinodal decomposition could result in periodic nanostructures^42–44^. Yong *et al*. used a phase-field model to simulate the formation of both lateral concentration modulations (LCMs) and vertical concentration modulations (VCMs) caused by spinodal decomposition during film deposition^45^. At a relatively slow growth rate, LCMs are easily developed; however, when the growth rate rises, LCMs tend to grow into VCMs. For the temporal evolution of the LCMs, over time, some neighboring twin lines would evolve into a “tuning fork” whereas three neighboring lines could evolve with the center line broken in several places where the left line and right line widen right below. The concentration modulations tend to grow continuously until a new equilibrium is reached^46^.

With the formation of the V-shaped features, the local Bi content is considerably higher in the V-shaped nanowalls than in other regions, which increases the risk of spinodal decomposition. The atomic size of the Bi atom is much larger than that of the P atom. The lattice strain is highest at the bottom of the valley of V-shaped nanowalls where phase separation is assumed to start. These decomposed Bi atoms are accumulated at the bottom of the valley of the V-shaped nanowalls to relax the local strain. Once spinodal decomposition occurs, LCMs, instead of VCMs, form because of the slow growth rate, resulting in the formation of Bi nanowalls. The periodic Bi-rich nanowalls grow toward (001) until the initial strain is compensated to a new equilibrium. The twin and triple Bi lines away from the InPBi/InP interface in the TEM are consistent with the reported LCM patterns that have evolved over time by simulation^45^.

### Effect on optical properties

The effect of the peculiar distribution of Bi in InPBi on the optical properties of InPBi, as observed by TEM and APT, is discussed in this section. The optical properties of InPBi is quite different from those of other dilute bismides. InPBi shows a broad below-bandgap PL spectrum at room temperature, which is attributed to two deep levels: a P_In_ antisite related donor level and a Bi-related acceptor level^26^.

**Figure 7:**
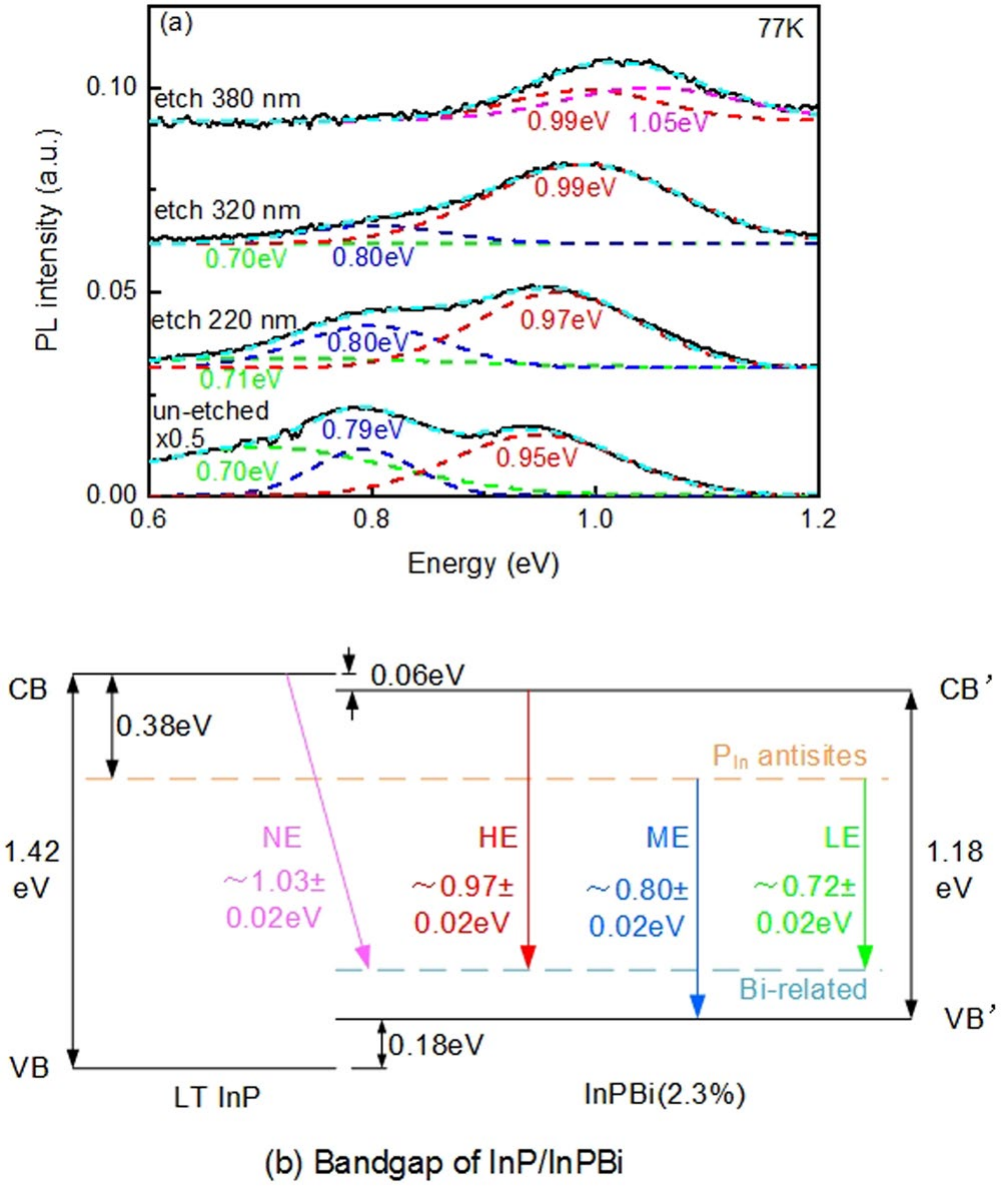
(**a**) Photoluminescence of InPBi at 77 K before and after etching. For the as-grown 220 nm and 320 nm etched specimens, the PL spectra consist of three peaks: low energy peak, middle energy peak and high energy peak at 0.72 ± 0.02 eV, 0.80 ± 0.02 eV and 0.97 ± 0.02 eV, respectively. For the 380 nm etched specimen, low and middle energy peaks disappear and a new peak at 1.05 eV emerges. (**b**) Bandgap diagram of InP/InPBi. A P_In_ antisite related deep level in LT-grown InP and InPBi and a Bi-related deep level in InPBi are observed. Four transitions referred to as LE, ME, HE and NE represent four different energy peaks in (**a**).

Specimen D was etched away at 220 nm, 320 nm and 380 nm, respectively, to investigate the effect of Bi distribution on PL. Figure 7(a) presents the PL spectra at 77 K of Specimen D before and after etching. The bottom curve labeled as “un-etched × 0.5” indicates that the intensity of this curve is multiplied by 0.5. According to deep-level transient spectroscopy (DLTS) results^26^, the PL spectrum consists of three peaks, high energy(HE), middle energy(ME) and low energy(LE) peaks. These peaks correspond to the recombination from the conduction band to the Bi-related level, the P_In_ antisite level to valance band and the P_In_ antisite level to the Bi-related level, respectively, as shown in Fig. 7(b). For InP_1−x_Bi_x_ with *x* = 2.3%, the HE, ME and LE was 0.97 ± 0.02 eV, 0.80 ± 0.02 eV and 0.72 ± 0.02 eV, respectively. For the three etched specimens, the HE peak is slightly blue-shifted. For the InP_1−x_Bi_x_specimen etched at 380 nm, the LE and ME peaks disappear and a new peak at 1.05 eV emerges. The shift in conduction band is predicted to be about −27 meV/%Bi for InPBi^7^, i.e., −0.06 eV in this case. The newly emerged peak, denoted as NE, is attributed to the recombination between the conduction band of InP and the Bi-related deep level.

**Figure 8:**
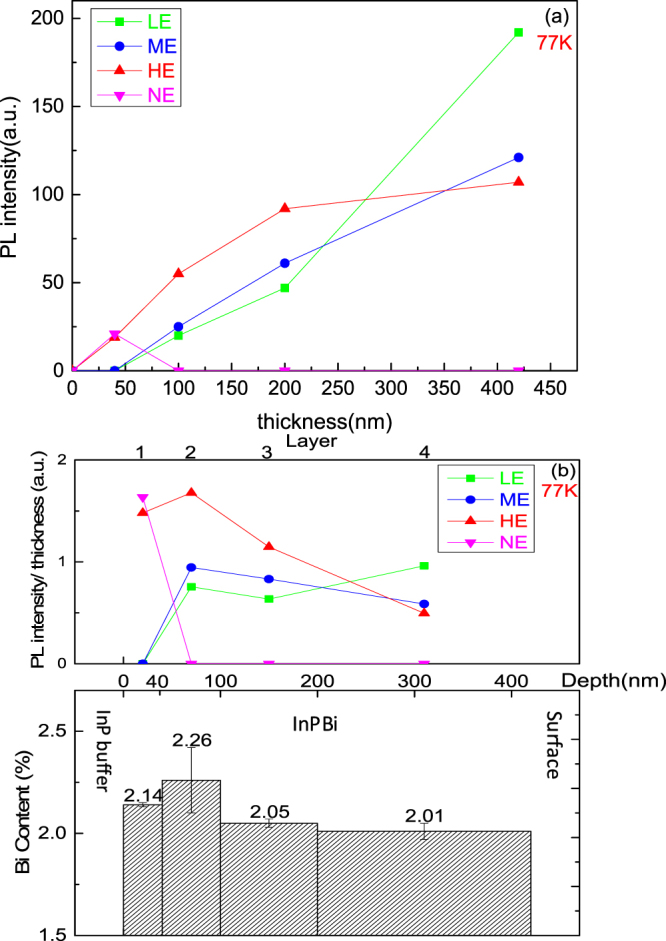
(**a**) PL intensity of different energy peaks as a function of the thickness of InPBi; (**b**) Average PL intensity per thickness of different energy peaks of InPBi on different layers, with the bottom graph depicting the average Bi content in different layers deduced from Fig. 3.

Table 1 presents the integrated PL intensity of the different peaks of the un-etched and etched InPBi specimens. The thickness of InPBi films is 420 nm; thus, we normalize the overall PL intensity to 420. According to the proportion of integrated PL intensity of the four different energy peaks, we could deduce the PL intensities for LE, ME, HE and NE as 192, 121, 107 and 0, respectively. Similarly, the PL intensities of the four peaks for the etched InPBi are deduced, as shown in Fig. 8(a).

**Table 1:** Proportion of the integrated PL of different peaks of un-etched and etched InPBi specimens.

Integrated PL	LE Peak	ME Peak	HE Peak	NE Peak
Un-etched × 0.5	45.58%	28.81%	25.61%	/
Etch 220 nm	23.67%	30.50%	45.83%	/
Etch 320 nm	19.50%	25.37%	55.13%	/
Etch 380 nm	/	/	46.96%	53.04%

To evaluate the correlation between the Bi distribution and the PL peaks, the InPBi film is divided into four parts. Layers 1, 2, 3 and 4 represent the InPBi layers measuring 0~40 nm, 40~100 nm, 100~200 nm and 200~420 nm, respectively, from the InP/InPBi interface to the surface. *D*_*i*_ is the measured integrated PL intensity of InPBi for layer *i* and all the InPBi layers situated below, whereas while ${D}_{i}^{^{\prime} }$ is the integrated PL intensity per thickness of InPBi for layer *i* with the same incident laser injected on the surface of layer *i*. With the absorption in InPBi considered, ${D}_{i}^{^{\prime} }$ can be calculated as follows:

1 $${D}_{i}^{^{\prime} }=\{\begin{array}{c}({D}_{i}-{D}_{i-1}{e}^{-\alpha {d}_{i}})/{d}_{i}(1-{e}^{-\alpha {d}_{i}}),\,2\le i\le 4\\ {D}_{i}/{d}_{i},\,i=1\end{array}$$

where α is the absorption coefficient of InPBi, assumed to be equal to that of InP, 9.689 × 10^4^ cm^−1^, at the incident photon energy of 2.3 eV^47^. *d*_*i*_ is the thickness of layer *i*. The calculated ${D}_{i}^{^{\prime} }$ of LE, ME, HE and NE peaks are shown in Fig. 8(b). The bottom graph in Fig. 8(b) depicts the average Bi content deduced from Fig. 3.

The overall PL intensities, including those from NE, HE, ME and NE are 3.12, 3.38, 2.61 and 2.04 for layers 1, 2, 3 and 4, respectively. With measurement error considered, the overall PL intensities for layers 1 and 2 are almost equal and continue to decrease from layer 3 to layer 4 because of photon reabsorption, which is stronger when the material is thicker. Compared with the PL between layers 1 and 2, the PL of NE exists in layer 1, whereas those of ME and LE exist in layer 2. The Bi contents are nearly equal in layers 1 and 2; however, the Bi-rich V-shaped nanowalls only appear on layer 1, with reference to the TEM image. This finding suggests that the Bi-rich V-shaped nanowalls hamper the recombination related to the P_In_ antisite level, causing the LE and ME peaks to disappear for reasons yet undetermined. Compared with that between layers 2 and 3, the PL intensity of HE sharply declines because of reabsorption and the decrease in Bi content; meanwhile, the PL intensities of ME and LE slightly fluctuate within the error range. The absorption coefficient is inversely proportional to the energy of exciting photons^47^; thus, the reabsorption of the excited photons from HE is significant, whereas that process of ME and LE is negligible in layer 3. As for layer 4, the PL intensity of HE continuously decreases but with a smaller reduction in slope under the competing effect of the vanishing of Bi content reduction and enlargement of reabsorption thickness. With an increase in the thickness of layer 4, the reabsorption of excited photons of ME becomes non-negligible, causing a reduction in PL intensity. Part of the reabsorbed photons from HE and ME are converted into LE, thereby increasing its PL intensity.

In conclusion, we observed anomalous distributions of Bi atoms in InPBi thin films, as confirmed by both APT and TEM with quasi-periodic Bi nanowalls in (1–10) plane. The plane stretches toward the surface from the center of Bi-rich V-shaped nanowalls in the (−111) and (1–11) planes at the InPBi/InP interface, which have not been observed in other dilute bismides. The V-shaped nanowalls at the InPBi/InP interface are attributed to Bi-induced droplet epitaxy, whereas the Bi nanowalls could be induced by spinodal decomposition. The optical properties of InPBi are strongly related to such Bi distributions, which affect the amount of the Bi-related deep level and particularly the Bi-rich V-shaped nanowalls that hamper the recombination related to the P_In_ antisite level.

## Methods

Samples were grown on semi-insulating (001) InP substrates by V90 gas source MBE. Phosphine was cracked at 1000 °C to form P_2_. Elementary In and Bi sources were used and fluxes were controlled and calibrated by adjusting the effusion cell temperatures. The InP substrate was first deoxidized at 545 °C, measured by a thermocouple with P_2_ flux impinged onto the substrate surface. Subsequently, a 69 nm thick undoped InP buffer layer was grown at 495 °C with a P_2_ pressure of 630 Torr. An InPBi thin film with a thickness of 420 nm was then grown at 345 °C with a P_2_ pressure of 350 Torr. The as grown InP_1−x_Bi_x_ had a Bi content of 2.3% measured by SIMS, which was calibrated based on Rutherford backscattering (RBS). All measured specimens were chosen from different positions of the same as-grown sample wafer.

Cross-section TEM specimen A was prepared using standard procedures, i.e., by mechanical lapping and dimpling followed by broad beam argon ion milling. The samples were investigated either with a JEOL 2100 F microscope operating at 200 kV or on a double spherical aberration corrected Titan Themis^3^ microscope operating at 200 kV. The crystal polarity of the TEM specimens were carefully determined using two independent methods: high-resolution Z-contrast imaging and convergent beam electron diffraction (CBED) techniques^48^. High-resolution energy dispersive X-ray spectrum image (EDX-SI) was acquired with the Titan system by using the Super-X detector at an extremely high probe current of about 1 nA and a reduced probe convergence angle of 18 mrad to enhance channeling. The Super-X detector comprises four silicon drift detectors (SDD) symmetrically placed around the optical axis, close to the sample area. All four signals are combined into one spectrum to improve the collection efficiency. Under these conditions, an EDX signal count rate of more than 85 kilo counts per second (kcps) at the specimen position was achieved. Data were acquired and quantified with FEI Velox software in which a standard Cliff-Lorimer (K-factor) quantification with absorption and geometry correction was implemented. The STEM-EDX maps of the specimen were collected within 30 min by drift correction.

Two specimens (Specimens B and C) were prepared and APT was performed by company CAMECA. The APT pillar specimens were prepared as follows: First, the specimens were coated with a protective GaAs capping layer on top. A lift-out wedge was then pulled and mounted onto specimen posts. The specimens were subsequently sharpened using annular ion mills in focused ion beam (FIB) to produce the final tips with a length of 1.8 μm, a bottom diameter of 380 nm and a tip diameter of 110 nm. These results were acquired under a laser energy of 0.05 pJ with a detection rate of 2% at a base temperature of 30 K.

After growth, Specimen D was etched using a mixed solution of HCl:H_3_PO_4_ (1:4) at room temperature; the etching rate was about 6.7 nm/s. Specimen D was etched away for 220 nm, 320 nm and 380 nm, respectively, to investigate the optical properties. The PL spectra were measured using a Fourier transform infrared (FTIR) spectrometry-based PL system in the rapid-scan mode rather than the step-scan mode in which a liquid-nitrogen cooled InSb detector and a CaF_2_ beam splitter were used. Laser with a wavelength of 532 nm was used as the excitation.

## Electronic supplementary material

Supporting information for: Nanoscale distribution of Bi atoms in InP1-xBix
