# Supplementary material for: Nanoscale distribution of Bi atoms in InP1−xBix
Source: Sci Rep. 2017 Sep 25;7:12278. doi: 10.1038/s41598-017-12075-2 (PMC5612989; doi:10.1038/s41598-017-12075-2)
Supplement: Supplementary file 1 — Supporting information for: Nanoscale distribution of Bi atoms in InP1-xBix [file 41598_2017_12075_MOESM1_ESM.pdf]

supporting information for:

Nanoscale distribution of Bi atoms in $InP_{1-x}Bi_x$

Liyao Zhang[1], Mingjian Wu[2,3], Xiren Chen[4], Xiaoyan Wu[1], Erdmann Spiecker[2], Yuxin Song[1], Wenwu Pan[1], Yaoyao Li[1], Li Yue[1], Jun Shao[4] and Shumin Wang[1,5,*]

[1]State Key Laboratory of Functional Materials for Informatics, Shanghai Institute of Microsystem and Information Technology, CAS, 865 Changning Road, Shanghai 200050, China

[2]Institute of Micro- and Nanostructure Research & Center for Nanoanalysis and Electron Microscopy (CENEM), Department of Materials Science, Universität Erlangen-Nürnberg, Cauerstraße 6, D-91058 Erlangen, Germany

[3]Paul-Drude-Institut für Festkörperelektronik, Hausvogteiplatz 5-7, D-10117 Berlin, Germany

[4]National Laboratory for Infrared Physics, Shanghai Institute of Technical Physics, CAS, 500 Yutian Road, Shanghai, 200083, China

[5]Department of Microtechnology and Nanoscience, Chalmers University of Technology, 41296 Gothenburg, Sweden

[*]Correspondence and requests for materials should be addressed to S. M. W (shumin@mail.sim.ac.cn)

STEM Z-contrast images

Experiments

The process of sample preparation for TEM measurement can be found in the paper. The high-angle annular dark-field (HAADF) STEM were acquired on the Titan system with a probe semi-angle of 24 mrad, and using a detector collection semi-angle of about 70–300 mrad, ensuring the incoherent Z-contrast imaging condition[1].

Results

Figure 1 shows STEM Z-contrast images at different magnifications. The incorporation of heavier Bi atoms is clearly seen by the brighter contrast in the epilayer. Bi-rich nano-walls about 4 nm is seen by the slightly brighter vertical stripes. From the intensity profile extracted from Fig. 1(a) and shown as inset, the peak is sided by valleys at both sides, consistent with APT analysis of the Bi profile. At a higher magnification, complete coherent interface between InPBi and InP is revealed. The sharp intensity increase crossing the interface indicate an atomically sharp interface. Interestingly, we found kinks of 6 double layer over a lateral distance of about 60 nm, as marked by the guidelines and arrows in Fig. 1(c).

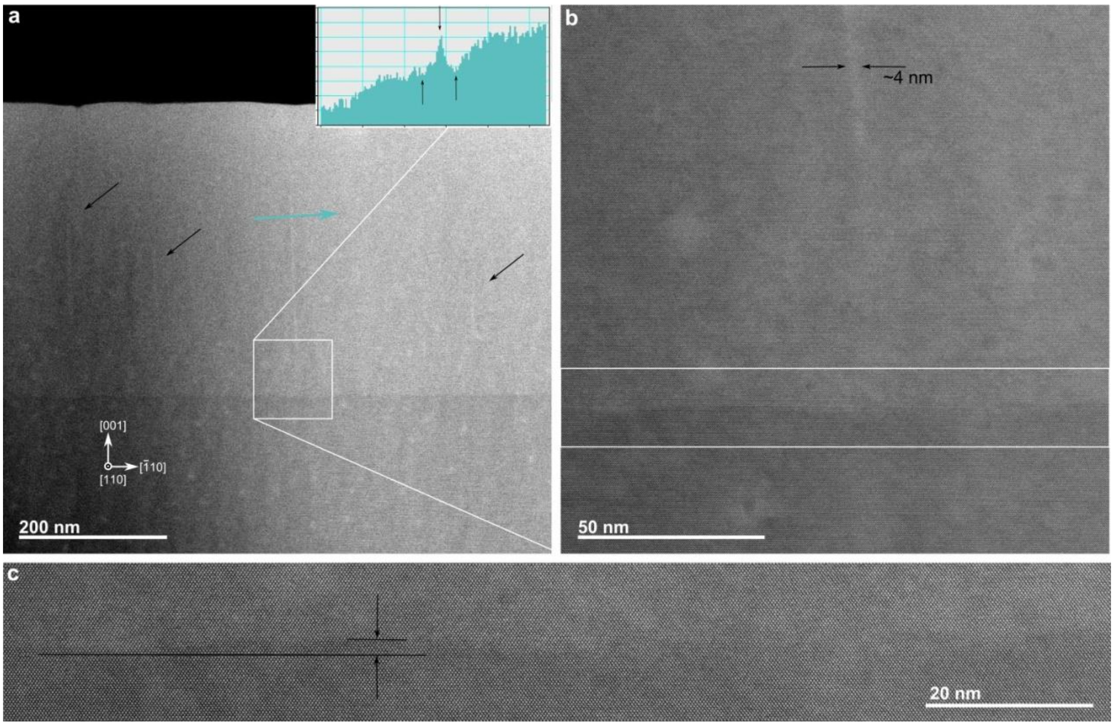

FIG. 1. STEM Z-contrast images at low (a), medium (b) and high (c) magnification. The nature of Bi-rich nano-wall of about 4 nm is evidenced by the brighter stripes in the image contrast, as marked by the black arrows. A line profile crossing one of such nano-wall is extracted and show as inset in (a). The InPBi/InP interface is clearly visible by the higher intensity in the Z-contrast images, and 5 to 6 atomic scale kinks of the InPBi/InP interface is evidenced in (c). The contrast change from darker left to brighter right in (a) is due to the change of sample thickness, and the spotty artifacts are due to preparation damage known[2] in InP samples.

Reference

[1] Stephen J. Pennycook and Peter D. Nellist. Scanning Transmission Electron Microscopy — imaging and analysis. Springer, 2011

[2] Arpad Barna and Bela Pecz. Simple Method for the Preparation of InP Based Samples for TEM Investigation. Journal of electron microscopy, 18:325–328, 1991.
